# Screen-based sedentary time: Association with soft drink consumption and the moderating effect of parental education in European children: The ENERGY study

**DOI:** 10.1371/journal.pone.0171537

**Published:** 2017-02-09

**Authors:** Mekdes K. Gebremariam, Mai J. Chinapaw, Bettina Bringolf-Isler, Elling Bere, Eva Kovacs, Maïté Verloigne, F. Marijn Stok, Yannis Manios, Johannes Brug, Nanna Lien

**Affiliations:** 1 Department of Nutrition, Faculty of Medicine, University of Oslo, Oslo, Norway; 2 Department of Public and Occupational Health, EMGO Institute for Health and Care Research, VU University Medical Center, Amsterdam, The Netherlands; 3 Department of Epidemiology and Public Health, Swiss Tropical and Public Health Institute, Basel, Switzerland; 4 University of Basel, Basel, Switzerland; 5 Department of Public Health, Sport and Nutrition, University of Agder, Kristiansand, Norway; 6 Department of Paediatrics, Medical Faculty, University of Pécs, Pécs, Hungary; 7 Institute for Medical Information Processing, Biometrics and Epidemiology German Centre for Vertigo and Balance Disorders, Ludwig Maximilian University, Munich, Germany; 8 Department of Movement and Sport Sciences, Ghent University, Ghent, Belgium; 9 Department of Psychological Assessment and Health Psychology, University of Konstanz, Konstanz, Germany; 10 School of Health Science & Education, Department of Nutrition and Dietetics, Harokopio University, Athens, Greece; 11 Faculty of Social and Behavioral Sciences, University of Amsterdam, Amsterdam, The Netherlands; National Institute for Agronomic Research, FRANCE

## Abstract

**Aim:**

The aim of the present study was to explore if children who spend more time on screen-based sedentary behaviors (i.e.TV viewing and computer use) drink more sugar-sweetened soft drinks. The study also assessed whether these associations were independent of individual and home environmental correlates of soft drink consumption and whether they were moderated by parental education.

**Methods:**

Data were collected from 7886 children participating in the EuropeaN Energy balance Research to prevent excessive weight Gain among Youth (ENERGY) survey conducted in eight European countries. Self-report questionnaires were used. Multilevel linear regression analyses with soft drink consumption as dependent variable, TV viewing and computer use as independent variables and age, gender, parental education, attitude towards soft drinks, self-efficacy, parental modelling, parental rules and home availability of soft drinks as covariates were conducted. Further interactions were tested to explore if these associations were moderated by parental education. Country-specific analyses were conducted.

**Results:**

In six of the eight included countries, a significant positive association was observed between TV viewing (min/day) and soft drink consumption (ml/day), independent of individual and home environmental correlates of soft drink consumption (B = 0.46 (0.26–0.66) in Greece, B = 0.77 (0.36–1.17) in Norway, B = 0.82 (0.12–1.51) in Hungary, B = 1.06 (0.67–1.46) in Spain, B = 1.21 (0.67–1.74) in Belgium and B = 1.49 (0.72–2.27) in Switzerland). There was no significant association between computer use and soft drink consumption in six of the eight included countries in the final models. Moderation effects of parental education in the association between TV viewing and soft drink consumption were found in Norway and Hungary, the association being stronger among those with low parental education.

**Conclusions:**

TV viewing appears to be independently associated with soft drink consumption and this association was moderated by parental education in two countries only. Reducing TV time might therefore favorably impact soft drink consumption.

## Introduction

Excessive consumption of sugar-sweetened beverages (SSB) has been found to be associated with overweight and obesity [1, 2]. Existing literature shows that screen-based sedentary behaviors (SB) (in particular TV viewing) are associated with SSB and other dietary behaviors among youth. Whereas associations with unhealthy dietary behaviors such as SSB consumption are often found to be positive, the association between SB and fruit/vegetable intake is found to be inverse [3–5]. This association between SB and dietary behaviors may be part of the reason why SB is a risk factor for unnecessary weight gain and overweight/obesity [6–10]. Several causal hypotheses have been put forward to explain how SB may influence dietary behaviors. One such hypothesis is the role of TV and online food advertisements for food choice and intake [11–14]. In Europe, a significant proportion of the food marketed to children is found to be unhealthy, the key promotional medium for such advertising being television [15]. Another potential mechanism linking SB to dietary behaviors is the role that SB such as TV, computer or electronic game use play as dishabituators or distractors [14, 16, 17]. This disruption of habituation to food cues might lead to overeating. Parents may also play a role in this association, by providing to their children energy dense foods and drinks during screen-viewing. SB repeatedly accompanied by the consumption of unhealthy food items might consequently become automatic cues to such dietary habits [18–20].

Most previous studies looking at the association between screen time and SSB consumption among youth have not considered the possible confounding role of important variables. Adjustment has often been limited to socioeconomic factors when exploring this association. However, it is possible that influences such as personal beliefs and attitudes, parenting style and other personal and home environmental variables related to dietary behaviors are responsible for the association between SB and dietary behaviors. As an example, it is possible that parents with stricter rules related to soft drink consumption also have stricter rules related to screen time. Favorable or unfavorable personal attitudes and beliefs related to one of the behaviors can also be related to similar attitudes and beliefs towards the other behavior. The present study will provide an opportunity to explore whether the association between SB and soft drink consumption is independent of personal and home environmental variables related to soft drink consumption.

Further, the literature shows that both screen time and soft drink consumption vary by socioeconomic position (SEP), being higher among those with a low SEP [21–23]. Children from a low socioeconomic background are also more likely to engage in eating/drinking when engaging in sedentary behaviors such as TV viewing than children from a high socioeconomic background [23]. A higher availability of unhealthy foods/drinks is also documented in families with low SEP [10], which might encourage overconsumption of such items during screen time [24]. It can consequently be hypothesized that parental education could moderate the association between SB and soft drink consumption, and that the association would be stronger among those with low parental education.

There are large differences in the consumption of soft drinks among youth across European countries including the countries participating in the present study [25]. There are also country-level differences in the magnitude of parental educational differences in SB and soft drink consumption [25]. In addition, some European countries have a ban on TV advertisements aimed at children, such as Norway where TV advertisements targeting children under 12 are banned. Other countries on the other hand have self-regulatory guidelines which might be less well reinforced [26]. These factors taken together might lead to differences between countries in the association between SB and soft drink consumption and in the moderating effects of interest.

Against this background, the study aimed to answer the following research questions: Are TV viewing and computer use associated with the consumption of soft drinks in European schoolchildren? Are the abovementioned associations independent of individual and home environmental variables related to soft drink consumption? Are the associations moderated by parental education? Are there any country-level differences in the associations?

## Materials and methods

### Design and sample

Data were obtained from the EuropeaN Energy balance Research to prevent excessive weight Gain among Youth (ENERGY) study. This school-based cross-sectional survey was conducted among 10–12 year-olds in eight European countries (Belgium, Greece, Hungary, the Netherlands, Norway, Slovenia, Spain and Switzerland). Data were collected between March and July 2010 except for Switzerland where data collection was conducted between June and December 2010. Samples from Greece, Hungary, the Netherlands and Slovenia were nationally representative. In Spain, Belgium, Norway and Switzerland schools were selected from the Aragon region, Flanders, Southern regions and German speaking regions respectively. A detailed description of the design and methodology of the survey has been published elsewhere [27].

Consent for participation was first obtained from school administrators. Thereafter, parents of participating children provided written informed consent. In the Netherlands, passive informed consent was allowed by the ethical committee, i.e. parents were informed and could opt out of the study. The study was approved by the relevant ethical committees in all participating countries: The Medical Ethics Committee of the Ghent University Hospital in Belgium; The Bioethics Committee of Harokopio University in Greece; The Scientific and Ethics Committee of Health Sciences Council in Hungary; The Medical Ethics Committee of the VU University Medical Center in The Netherlands; The National Committees for Research Ethics in Norway; The National Medical Ethics Committee of the Republic of Slovenia; The Clinical Research Ethics Committee of the Government of Aragon in Spain and the ethics committees of the participating cantons in Switzerland.

### Data collection

Pen and paper questionnaires were used to collect data from the children during school time. Detailed information on the test-retest reliability and construct validity of the questionnaire items has been published elsewhere [28]. A total of 730 children were included in the test-retest study and 96 were included in the construct validity study conducted in six countries. Good test-retest reliability and moderate to good construct validity were obtained for the large majority of items used, except for children’s attitude toward soft drink consumption and parental allowance of soft drink consumption for which validity was poor [28]. Parental education was reported by one of the parents via a questionnaire.

### Measures

#### Outcome measure: Soft drink consumption

Soft drink consumption was assessed using a frequency question (with seven response categories ranging from never to more than once every day). In the introductory text to these questions, soft drinks were explained as fizzy drinks (e.g. cola) and fruit squash (e.g. ice tea) but not diet drinks and 100% fruit juice. Sports and energy drinks (e.g. lucozade, relentless) were also included. The amount consumed was assessed by asking the children to indicate the number of glasses or small bottles (250 ml), cans (330 ml) and large bottles (500ml) they drink. Frequency and amount were multiplied to get a daily consumption measure (ml/day). The measures had acceptable test-retest reliability (ICC≥0.53) [28].

#### TV viewing and computer use

Time spent watching TV (including video and DVDs) was assessed separately for weekdays and weekend days with nine answer categories ranging from “no viewing” to “4 hours or more per day”. Computer use (games, leisure activities) was measured in a similar manner. The mean TV time and computer time in hours per day was calculated. The measures had good test-retest reliability (ICC = 0.67 and 0.68) [28].

#### Parental education

Parents reported their own level of education, as well as the level of education of the other parent/caregiver, using the following answer categories: less than 7 years; 7–9 years; 9–11 years; 12–13 years; 14 years or more. As educational systems differ considerably across Europe, the number of years of formal education was used as an indicator for level of education. Maternal and paternal education levels were combined and the variable was categorized as being high (i.e. at least one parent more than 14 years of education) or low (i.e. both parents less than 14 years of education). This categorization approximately distinguishes families with at least one caregiver who has completed medium or higher vocational, college or university training from other families.

#### Correlates of soft drink consumption

Children’s attitude related to soft drink consumption was assessed using the question: “I think that drinking fizzy drinks or fruit squash is…” using a 5-point scale with answer categories ranging from “very bad” to “very good” and a neutral mid-point. Children’s self-efficacy related to the consumption of soft drinks was assessed using the question: “I find drinking no fizzy drinks or fruit squash” using a 5-point scale with answer categories ranging from “very difficult” to “very easy”.

Parental modelling was assessed using the question: “How often do your parents/care givers drink fizzy drinks or fruit squash?” Parental allowance of soft drink consumption was assessed using the question: “I am allowed to take fizzy drinks or fruit squash, whenever I want.” Home availability of soft drinks was assessed using the question “Are there usually fizzy drinks or fruit squash at your home?” These three questions had answer categories ranging from “Never” to “Always”.

These correlates were chosen for inclusion in the analyses because of their significant associations with soft drink consumption and TV/computer use in the whole sample (results not shown).

Test-retest reliability for these variables was moderate to high (ICC = 0.55–1.00) [28].

### Statistical analysis

Descriptive analyses were first conducted (Table 1). Due to the clustering of students at the school level within each country, two-level multilevel regression analyses were then conducted. First, the association between [1] TV viewing and [2] computer use and the consumption of soft drinks was explored, with soft drink consumption as dependent variable and the SB as independent variables, adjusting for age, gender, parental education and the SB not used as independent variable (Model 1, Table 2). Individual correlates of soft drink consumption (attitude related to soft drink consumption and self-efficacy towards reducing soft drink consumption) were then added to Model 1 (Model 2, Table 2). Thereafter, home environmental correlates (parental modelling, parental rules and home availability of soft drinks) were added to Model 2 (Model 3, Table 2).

**Table 1:** Total and country-specific characteristics of the study sample (n = 5710).

	Age (SD)	Gender (% girls)	Parental education (% low)	Soft drink intake (ml/day)	TV viewing (min/day)	Computer use (min/day)
Total	11.6 (0.7)	53	35	303 (454)	108 (60)	75 (56)
Belgium (n = 666)	11.5 (0.7)	55	16	384 (493)	110 (61)	72 (53)
Greece (n = 891)	11.3 (0.6)	55	48	102 (174)	124 (57)	71 (56)
Hungary (n = 763)	12.2 (0.6)	57	42	547 (621)	120 (62)	93 (62)
Netherlands(n = 349)	11.6 (0.7)	51	22	533 (521)	102 (63)	86 (63)
Norway (n = 718)	12.0 (0.7)	52	26	219 (285)	99 (52)	77 (55)
Slovenia (n = 897)	11.4 (0.6)	53	44	295 (470)	113 (63)	76 (61)
Spain (n = 880)	11.4 (0.6)	51	19	181 (304)	103 (55)	72 (55)
Switzerland (n = 546)	11.6 (0.8)	49	60	369 (502)	78 (54)	51 (47)

Results presented as mean (SD), parental education was defined as low when both parents had less than 14 years of education; it was defined as high when at least one parent had more than 14 years of education

**Table 2:** Association between soft drink consumption (ml/day) and TV/computer use (min/day) among study participants.

	Model 1^$^ (Est. (CI))	Model 2^#^ (Est. (CI))	Model 3^¤^ (Est. (CI))
Belgium (n = 666)			
TV	1.93 (1.32–2.53)***	1.39 (0.83–1.95)***	1.21 (0.67–1.74)***
Computer	1.55 (0.84–2.26)***	0.56 (-0.10–1.23)	0.11 (-0.53–0.76)
Greece (n = 891)			
TV	0.67 (0.46–0.87)***	0.57 (0.37–0.77)***	0.46 (0.26–0.66)***
Computer	0.31 (0.10–0.52)**	0.24 (0.03–0.45)*	0.12 (-0.09–0.33)
Hungary (n = 763)			
TV	2.07 (1.32–2.82)***	1.75 (1.02–2.48)***	0.82 (0.12–1.51)*
Computer	1.60 (0.83–2.37)***	1.12 (0.37–1.87)**	1.01 (0.31–1.71)***
Netherlands (n = 349)			
TV	- 0.22 (-1.31–0.86)	-0.40 (-1.40–0.60)	-0.56 (-1.53–0.41)
Computer	1.26 (0.19–2.34)*	0.002 (-1.05–1.05)	-0.32 (-1.33–0.69)
Norway (n = 718)			
TV	1.14 (0.71–1.57)***	0.88 (0.47–1.29)***	0.77 (0.36–1.17)***
Computer	0.67 (0.27–1.12)***	0.50 (0.08–0.91)*	0.35 (-0.05–0.75)
Slovenia (n = 897)			
TV	1.10 (0.54–1.67)***	0.81 (0.27–1.35)**	0.49 (-0.06–1.03)
Computer	1.35 (0.76–1.93)***	0.99 (0.42–1.56)**	0.79 (0.24–1.36)***
Spain (n = 880)			
TV	1.47 (1.07–1.87)***	1.29 (0.89–1.68)***	1.06 (0.67–1.46)***
Computer	0.46 (0.06–0.87)*	0.36 (-0.03–0.76)	0.23 (-0.16–0.62)
Switzerland (n = 546)			
TV	2.65 (1.80–3.50)***	2.17 (1.35–2.99)***	1.49 (0.72–2.27)***
Computer	0.69 (-0.30–1.68)	0.44 (-0.52–1.40)	0.33 (-0.57–1.23)

Dependent variable in the regression analyses = soft drink consumption; independent variable = TV or computer use.

***p<0.001,

**p<0.01,

*p<0.05

^$^Model 1 was adjusted for age, gender, parental education and the other sedentary behavior

^#^Model 2 = Model 1 + individual correlates of soft drink consumption (attitude and self-efficacy)

^¤^Model 3 = Model 2 + home environmental correlates of soft drink consumption (parental modelling, parental rules and availability at home)

To assess the moderating effect of parental education in the association between [1] TV and [2] computer use and soft drink consumption, interaction terms (parental education*TV viewing and parental education*computer use) were entered in regression models. When significant moderation effects were detected, analyses were stratified by parental educational level (results presented in text).

In order to account for correlations between TV viewing and computer use, these variables were mutually adjusted for in the regression models as mentioned above. Due to the large differences in soft drink consumption between countries, only country-specific regression analyses were conducted. Assumptions for the regression analyses were checked and met.

Differences between included and excluded subjects were assessed using t-test and chi-squared test. All analyses were conducted using SPSS version 22 and the significance level was set to 0.05.

## Results

Participants with no data on parental education (parental non-participation (n = 1403) and missing data on parental education (n = 773)) were excluded from the analyses. An additional 29 participants had no information about any of the correlates included and were also excluded. The sample size for the present study was n = 5710.

Participants excluded from the study due to lack of parental data (non-response and missing data) were more likely to be males. They also had a significantly higher consumption of soft drinks and spent more time on TV viewing and computer use (data not shown).

Table 1 describes the characteristics of the included children. The average consumption of soft drinks was 303 ml per day. There were large variations in the consumption of soft drinks between countries, the lowest consumption being in Greece (102 ml/day) and the highest in Hungary (547 ml/day). The children spent on average 108 minutes/day watching TV and 77 minutes/day using the computer for games and leisure activities, with some differences between countries. The proportion of children with low educated parents was 35% for the total sample in this study.

### Associations between sedentary behaviors (TV viewing and computer use) and soft drink consumption

Table 2 shows the results of the regression analyses. After adjustment for age, gender and parental education, a statistically significant positive association was found between TV viewing (min/day) and soft drink consumption (ml/day) in all countries except the Netherlands. There was also a statistically significant positive association between computer use and soft drink consumption in all countries except Switzerland.

These analyses were subsequently adjusted for individual and home environmental correlates of soft drink consumption. The associations between TV viewing and soft drink consumption were attenuated but remained significant in six of the countries (B = 0.46 (0.26–0.66) in Greece, B = 0.77 (0.36–1.17) in Norway, B = 0.82 (0.12–1.51) in Hungary, B = 1.06 (0.67–1.46) in Spain, B = 1.21 (0.67–1.74) in Belgium and B = 1.49 (0.72–2.27) in Switzerland). In Slovenia, the association became non-significant once adjusted for individual correlates of soft drink consumption. The association remained insignificant in the Netherlands.

The association between computer use and soft drink consumption became non-significant in the Netherlands, Belgium and Spain after adjustment for individual correlates of soft drink consumption. In Greece and Norway, the associations remained became insignificant in the final model. The association between computer use and soft drink consumption was attenuated but remained significant in Slovenia (B = 0.79 (0.24–1.36)) and Hungary (B = 1.01 (0.31–1.71)).

### Moderating effect of parental education

A moderating effect of parental education in the association between TV use (min/day) and soft drink consumption (ml/day) was found in Hungary and Norway after adjustment for age and gender (B [TV viewing x parental education]) = -1.74 (-3.11, -0.37) in Hungary and B = -1.12 (-2.02, -0.23) in Norway with low education as reference category). The positive association between TV viewing and soft drink consumption was strongest among those with low parental education compared to those with high parental education: B = 3.09 (1.88–4.30), B = 1.37 (0.42–3.32) in Hungary; B = 1.96 (1.07–2.86); B = 0.87 (0.38–1.36) in Norway. A moderating effect of parental education in the association between computer use and soft drink consumption was found in Spain (cross-over interaction, i.e. different directions in the different parental educational groups). Accordingly, computer use was inversely associated with soft drink consumption in those with low parental education (B = -1.24 (-2.35, -0.13)), and positively related to soft drink consumption in those with high parental education (B = 0.96 (0.55, 1.37)). These moderation effects remained significant in the final models.

## Discussion

The present study explored the association between SB (TV viewing and computer use) and soft drink consumption among European schoolchildren. It assessed whether this association was independent of individual and home environmental correlates of soft drink consumption. The moderating effect of parental education in this association was also investigated. In six of the eight included countries, there was a significant positive association between TV viewing and soft drink consumption, independent of individual and home environmental variables related to soft drink consumption (not found in Slovenia and the Netherlands). An independent association between computer use and soft drink consumption was found only in Slovenia and Hungary. Moderation effects of parental education in the association between TV viewing and soft drink consumption were found in two of the eight included countries (Hungary and Norway), the associations being stronger in those with low parental education.

The positive association between TV viewing and soft drink consumption documented in this study is in line with findings in the literature [3, 4]. In the present study, adjustments were made for personal and home environmental correlates of soft drink consumption. The association between TV viewing and soft drink consumption, although attenuated, remained significant, indicating that it was independent of these correlates. Other mechanisms such as exposure to advertisements and habitual linking of TV viewing with consumption of soft drinks may therefore also play a role in this association. There might however also be residual confounding due to other factors not controlled for in this study. In a recent study among younger children aimed at assessing the confounding role of parental norms in the association between screen time and SSB consumption, screen time was found to predict SSB consumption independent of parental norms [29].

The magnitude of the association between TV viewing and soft drink consumption found in the present study was modest at best. An increase in TV viewing by an hour per day was associated with the consumption of 30 ml per day more soft drinks in Greece and 90 ml per day more soft drinks in Switzerland. In an age of rapidly developing technology where both screen-based devices as well as programs are becoming more numerous and appealing, children are likely to increasingly spend a significant time in sedentary pursuits. Therefore such an association remains important. It is also worth acknowledging the presence of measurement errors in exposure variables which could attenuate the strength of the association. Therefore, the associations may be stronger than reported [30]. The differences in the magnitude of this association between countries could, among other things, be due to differences in the exposure to advertisements on TV, as hypothesized in this study. In this regard, regulations related to advertisements targeted at children vary between European countries, from a complete ban during children’s TV programs in countries such as Norway to self-regulatory guidelines in other countries that may not always be well reinforced [26]. The lack of association between TV viewing and soft drink consumption in the Netherlands might be due to the fact that there is an over-representation of children whose parents have a high education.

The association between computer use and soft drink consumption became insignificant in five of the included countries after adjustment for correlates of soft drink consumption. This is in contrast to the findings of several other studies among children where a positive association is documented [3, 4]. However, among the studies of children and adolescents included in the recently published systematic review on this subject, none had adjusted for factors other than sociodemographic characteristics and body weight [4]. The present study therefore adds to existing knowledge by indicating that the association between computer use and soft drink consumption is not independent of personal and home environmental variables in most of the included countries. Future observational studies and in particular cross-sectional studies should adjust for such important variables in order to adequately understand the independent association between SB and dietary behaviors. There might be several reasons why TV viewing, and not computer use, is associated with soft drink consumption in several countries. As compared to computer use, TV viewing is related to more exposure to advertisements for energy dense foods and drinks. In addition, passive overconsumption of foods/drinks is more likely to occur during TV viewing than computer use, in particular when the latter involves activities such as games.

Identifying differences in the magnitude or direction of associations between correlates and dietary behaviors in different socioeconomic subgroups would inform our understanding of why socioeconomic differences in dietary behaviors occur. The moderating effect of parental education in the association between SB and the consumption of soft drinks was therefore explored in the present study. Such a moderating effect was found in Norway and Hungary, where the association as hypothesized was stronger among those with low parental education. Differences between subgroups in personal and home environmental factors contributing to the association between the behaviors could help explain this moderating effect. Eating and drinking while watching TV for example is found to be more common among children of parents with low education [23]. Another possibility is that the impact of advertising might differ for children from different socioeconomic backgrounds. A previous study among 4–12 year-olds found that the association between advertising exposure and overall food consumption was significant in low income families only [31]. This could reflect a higher resistance to the pressure to buy advertised foods by parents with a high socioeconomic background. This is however unlikely to explain the moderating effect in Norway where exposure to TV advertisements is limited. In several of the countries where TV viewing was associated with soft drink consumption, no such moderating effect was detected, although associations mostly tended to be stronger in those with low parental education. This suggests that in these countries, the mechanisms linking TV viewing and soft drink consumption are not affected by parental education to a significant degree. The differences in moderating effects between countries might reflect context-specific differences in the impact of parental SEP on adolescent health behavior.

The present study should be seen in light of the following weaknesses: the cross-sectional nature of the study does not allow for any causal inference to be made, although it is unlikely that soft drink consumption would affect sedentary time. The use of self-reported measures can lead to bias due to recall as well as social desirability, limiting validity and reliability, in particular in younger children. Nevertheless, there was a good test-retest reliability of the measures used in this study and moderate to good validity. Participants that were excluded from the analyses because of missing data on parental education had a higher consumption of soft drinks and higher TV viewing and computer time, and therefore might have been most likely from a lower socioeconomic background than the included participants. This might have affected the results obtained, in particular the results of the moderation analyses especially in countries where the parental non-response rate was high and might limit the external validity of the study. The present study was conducted in 2010. Since that period, significant changes in the availability of different screens have occurred (e.g. increased use of tablets, mobile phones), as well as changes in the mediums and methods used to access materials such as movies (e.g. online video streaming, time-shifted TV viewing). These changes might result in an increase in the time spent on screen-based SB. They might also result in changes in children’s exposure to advertising which might in turn affect the association between screen-based SB and dietary behaviors such as soft drink consumption. The present study included traditional screen-based SB only. The hypotheses linking TV viewing with dietary behaviors such as the disruption of habituation to food cues, the automatic linking of the SB with the consumption of some foods and exposure to advertisement might not apply to the same extent to other SB such as mobile phone use.

The strengths of the study include the large multinational sample of children participating in the survey. The inclusion of several individual and familial correlates of soft drink consumption allowed for adjustment of these factors thereby contributing to new knowledge in the area.

## Conclusion

TV viewing was positively associated with the consumption of soft drinks in six out of eight European countries; Slovenia and the Netherlands being the exceptions. Moderating effects of parental education in this association were found in two countries only. In six of the eight included countries, there was no significant association between computer use and the consumption of soft drinks after adjusting for individual and home environmental correlates of soft drink consumption. Reducing TV viewing behavior might therefore have a favorable impact on the consumption of soft drinks.

## Supporting information

S1 Dataset (SAV)
